# AIEgen-Based Fluorescent Nanomaterials: Fabrication and Biological Applications

**DOI:** 10.3390/molecules23020419

**Published:** 2018-02-14

**Authors:** Hui Gao, Xin Zhao, Sijie Chen

**Affiliations:** 1 Ming Wai Lau Centre for Reparative Medicine, Karolinska Institutet, Hong Kong, China; hui.gao.1@ki.se; 2 Department of Biomedical Engineering, The Hong Kong Polytechnic University, Hong Kong, China; xin.zhao@polyu.edu.hk

**Keywords:** fluorescent nanomaterials, AIE, bioimaging, cell tracing, theranosis

## Abstract

In recent years, luminogens with the feature of aggregation-induced emission (AIEgen) have emerged as advanced luminescent materials for fluorescent nanomaterial preparation. AIEgen-based nanomaterials show enhanced fluorescence efficiency and superior photostability, which thusly offer unique advantages in biological applications. In this review, we will summarize the fabrication methods of AIEgen-based nanomaterials and their applications in in vitro/in vivo imaging, cell tracing, photodynamic therapy and drug delivery, focusing on the recent progress.

## 1. Introduction

Fluorescent nanomaterials have been widely studied in recent years, due to their advantages of good brightness, excellent photostability, great flexibility in synthesis and functionalization, and their great potential in biological applications [1,2]. Organic dye-based fluorescent nanomaterials are some of the most popular fluorescent nanomaterials used in biological research. They show better biocompatibility compared to the nanomaterials based on inorganic semiconductor quantum dots (QDs), higher brightness compared to gold nanoparticles (NPs), etc. Moreover, their emission properties can also be easily manipulated by incorporating different kinds and amounts of dye molecules into matrixes [2].

**Figure 1:**
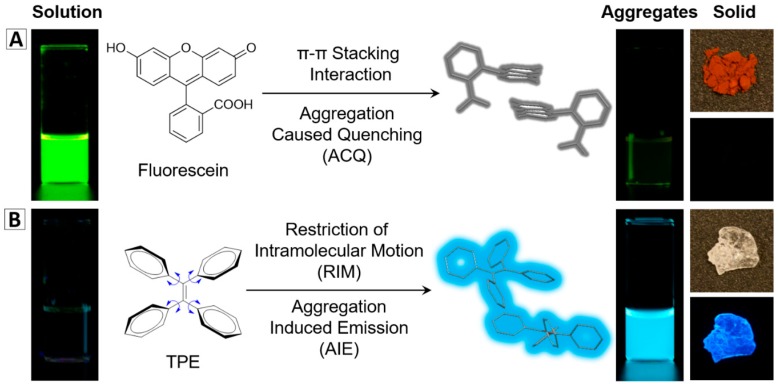
Typical example of (**A**) conventional fluorescent agents: fluorescein is a strong emitter in the solution state, but is quenched in the aggregate state or solid state; (**B**) AIEgens: tetraphenylethene (TPE) turns on its emission upon aggregate formation [2] (reproduced from [2] with permission (2016) of the Royal Society of Chemistry).

It is common knowledge that most organic fluorophores show good emission in dilute solutions. However, their emissions will be weakened or completely quenched when they aggregate in solid state or at a high concentration because of the strong π−π interactions. This phenomenon is called the aggregation-caused quenching (ACQ) effect (Figure 1A) [2]. Within an NP, dye molecules are incorporated into a small volume. Limited amount of dyes can be doped into one NP to avoid the fluorescence concentration quenching. The ACQ property of conventional dyes is thus a great hindrance for the development of bright fluorescent NPs [2,3,4].

The discovery of the AIE phenomenon opens a new avenue for the development of fluorescent materials and fluorescence-based technology [2,5,6,7]. The AIE molecules are almost non-emissive when molecules are dissolved but are bright emitters in the solid state or aggregate state as shown in Figure 1B. Rotational and/or vibrational motions of molecules can significantly consume excited-state energy, which are associated with non-radiative excited-state decay processes. The restriction of these intramolecular motions in the aggregate state blocks the non-radiative decay and results in the emission turn-on, finally leading to the AIE phenomenon [8]. Additionally, the non-planar conformations of the AIE molecules prevent π−π interactions [2,8,9]. Therefore, once incorporated into solid matrixes (e.g., solid NPs), the AIE molecules are fixed and thereby could ensure a bright emission for the formed NPs. The unique features of AIEgens make them good candidates for fabricating highly emissive fluorescent nanomaterials. To date, different series of AIEgen-based fluorescent nanomaterials have been successfully developed and used in biological research [2,9,10,11,12,13,14,15,16,17,18,19]. In this review, we will mainly discuss the fabrication of fluorescent nanomaterials based on AIEgens, and their biological applications in bioimaging, cell tracing, drug delivery, and cancer therapy.

## 2. Synthesis and Properties of AIEgen-Based Nanomaterials

### 2.1. Examples of AIEgens Used for Fluorescent Nanomaterials Fabrication

**Figure 2:**
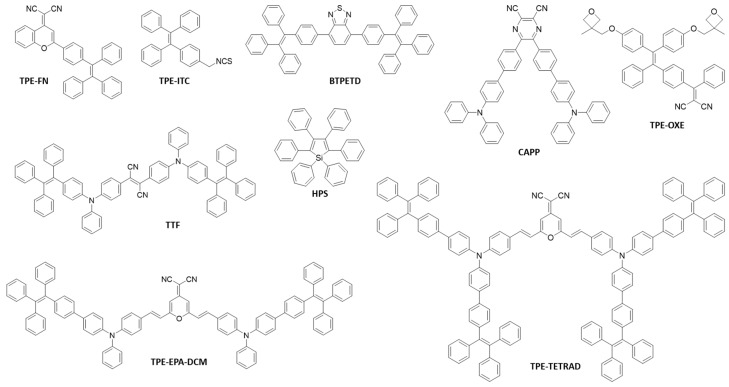
Typical AIEgens employed in nanomaterials fabrication.

AIEgens with different skeleton structures, peripheral groups and functional groups have been developed [1,20,21,22]. Figure 2 shows some examples of the AIEgens that are commonly employed in the fabrication of AIEgen-based fluorescent nanomaterials.

These molecules exhibit very low luminescence as molecular species, but are ultra-emissive with a high quantum yield (up to unity) in the aggregate or solid states [1,20,21,22,23]. In addition, they usually possess large Stokes shifts (≥100 nm) [24,25], which would avoid self-absorption and consequently benefit the imaging applications. The optical properties of some typical AIEgens are listed in Table 1. The AIEgens can be well-designed with different emission colors, tuning from blue (e.g., TPE [26], 1-[4-(1,2,2-Triphenylvinyl)phenyl]pyrene (TPEPy) [21]) to green (e.g., 1,1,2,3,4,5-hexaphenylsilole (HPS) [22], BTPETD [27]), and even red or near-infrared (NIR) (e.g., TPE-EPA-DCM [20], TTF [28]).

### 2.2. Fabrication of AIEgen-Based Nanomaterials

Due to their superior features compared to conventional fluorescent agents, AIEgens have been referred to as a type of advanced fluorescent agents for the design and preparation of fluorescent nanomaterials. The fluorescence intensity of AIEgens could be enhanced when they are entrapped and fixed in the solid nanomaterials. To date, various methods have been proposed to fabricate AIEgen-doped nanomaterials. They can be classified into physical and chemical method, in which the AIEgens are incorporated into the nanomaterials through non-covalent binding or covalent binding, respectively. Functionalization of these nanomaterials is discussed in the next section.

#### 2.2.1. Non-Covalent Binding

##### Amorphous AIE NPs

**Figure 3:**
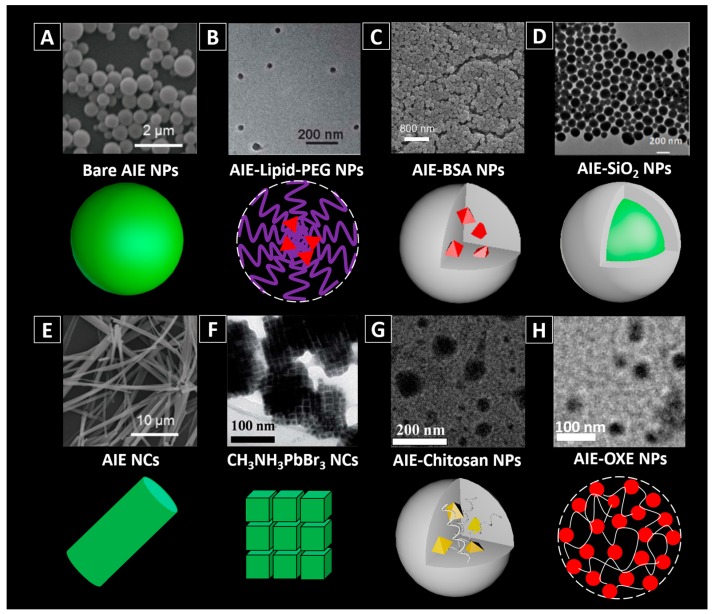
Different types of AIEgen-based nanomaterials. (**A**) bare AIE NPs [2,29]; (**B**) AIE-Lipid-poly(ethylene glycol) (PEG) NPs [2,24]; (**C**) AIE-BSA NPs [20]; (**D**) AIE-SiO_2_ NPs [2,34]; (**E**) bare AIE NCs [29]; (**F**) CH_3_NH_3_PbBr_3_ nanocrystals [38]; (**G**) AIE-Chitosan NPs [2,40]; (**H**) AIE-OXE NPs after photo-crosslinking [12]. Reprinted with permission from [2,20,24,29,34,38,39]. Copyright 2016 the Royal Society of Chemistry, Copyright 2012 Wiley-VCH, Copyright 2012 The Royal Society of Chemistry, Copyright 2017 Wiley-VCH, Copyright 2017 The Royal Society of Chemistry, Copyright 2017 Wiley-VCH, Copyright 2017 The Royal Society of Chemistry and Copyright 2016 Elsevier, respectively.

Some AIE dyes can be facilely assembled into NPs [29,30]. For example, 2-(2-(4-(1,2,2-triphenyl-vinyl)phenyl)-4*H*-chromen-4-ylidene)malononitrile (TPE-FN) aggregates with diameters of ~500 nm are formed by injecting a TPE-FN/tetrahydrofuran (THF) solution into DI-water under ultrasonication (Figure 3A) [29].

Some AIEgens such as TTF (an adduct of tetraphenylethene, triphenylamine and fumaronitrile), Figure 2, cannot self-assemble into NPs in buffer with high water content, due to their high hydrophobicity [2]. They would form into large precipitates rather than nano-aggregates when added into water. In this case, other approaches need to be used to achieve the controllable assembly of them into NPs. Nanoprecipitation, a common technique used for encapsulating hydrophobic drugs into the NPs, has been utilized in preparing AIEgen-loaded NPs. Usually, the hydrophobic AIEgens and the chosen amphiphilic molecules are first mixed with organic solvent and then introduced into water. Based on the theory that similarities can be solvable easily in each other, when poured into water, the hydrophobic parts of the amphiphilic molecules would prefer to cluster as the core, while their hydrophilic ends would expose to water and form a uniform shell (Figure 3B,C). For the lipophilic AIEgens, they are likely to stay in the hydrophobic core. Details and examples of such methods can be found in various references [16,25,28,31,32]. Natural polymers such as bovine serum albumin (BSA) [20,33] are also used to physically incorporate the AIEgens into fluorescent NPs via nanoprecipitation method. For example, TPE-EPA-DCM is successfully introduced into BSA (Figure 3C) via desolvation technique assisted precipitation method, forming TPE-EPA-DCM based BSA composite NPs, with diameter of ~100 nm.

AIEgens can also be incorporated into inorganic matrixes via non-covalent binding, such as coprecipitation method, sol-gel method, etc. [34,35]. For example, silica is one type of commonly used matrix materials, in which AIEgens can be incorporated facilely during the hydrolysis process of tetraethyl orthosilicate (TEOS) under well-controlled conditions [36]. Xiang and co-authors encapsulated AIEgens within silica matrix based on salicylaldehyde hydrazones through sol-gel reaction using TEOS as a silica source, and the formed NPs (AIE-SiO_2_) were afforded with bright fluorescence (Figure 3D) [37]. These AIEgen-doped silica NPs were found with much brighter fluorescence than the commercial fluorescein-doped silica NPs.

##### AIE Nanocrystals

Compared with the amorphous state, materials in crystallized state are more compact. For the AIEgens, crystallization could be an effective strategy to increase the brightness as it will minimize the intramolecular motions of the molecules [23,29,37]. People have observed formation of highly emissive organized nanostructures from silole derivatives [40], but their bio-applications have remained largely unexplored. Recently, Liu, et al. [29] investigated the crystallization effect on pure AIEgens. TPE-FN was employed to produce the amorphous aggregates and crystals. To enable small size and bright crystals for biological applications, they used a bottom-up method to fabricate nanocrystals using probe ultrasonication as the source of mechanical stress. In detail, a crystal seed suspension was pre-fabricated by injecting TPE-FN (in THF) into THF/water mixture at *f_w_* = 65% in a vial. Then the vial was tightly covered and placed in dark at room temperature for enough time to make sure that the crystallization was completed. While being sonicated, extra TPE-FN was introduced into deionized water (DI-water), and followed by this, the crystal seed suspension and additional THF was added to the system. After 60 s of crystallization, the mixture was dialyzed against DI-water to remove THF and the nanocrystals were obtained. Their study suggested that TPE-FN nanocrystals (Figure 3E) are more emissive compared with the amorphous aggregates made of the same dye molecules (Figure 3A), because crystallization can effectively minimize the intramolecular motions.

Interestingly, AIEgens have also been reported to be used to activate the assembly of semiconductor nanocrystals (NCs) with superstructure. Zhang and Dong applied a TPE derivative, 3-(4-(1,2,2-triphenylvinyl) phenoxy)propan-1-amine, to replace octylamine to fabricate CH_3_NH_3_PbBr_3_ nanocrystals [38]. As shown in Figure 3F, the CH_3_NH_3_PbBr_3_ nanocrystals are nanocubes (~11.1 nm) and are likely to assemble into ordered superstructures with the assistance of AIEgens. They proved that the assembly of the CH_3_NH_3_PbBr_3_ nanocrystals was correlated with the interactions between the TPE groups. Hence adjusting the chain length of the TPE derivative mattered the final results of the NCs, which may potentially be utilized for the control of their properties. 

#### 2.2.2. Covalent Binding

Dye loading based on non-covalent approaches is convenient and has less requirements for dye modification. On the other hand, covalent methods involve chemically bonded tethers showing a better control of the dye loading ratio in the nanomaterial fabrication process, and the covalently linked dye molecules inside the nanomaterials will not easily leak out [2].

To prepare AIEgen-based nanomaterials through covalent binding, people can first covalently link the functionalized AIEgens to polymers with corresponding reactive groups and use modified polymers to fabricate AIEgen-based nanomaterials, Figure 3G,H. [41,42,43] For example, the isothiocyanate group on TPE-ICT can react with the amino group on chitosan (CS), a natural biopolymer, via Schiff’s base reaction, Figure 3G [39,44]. For instance, Wang and co-authors have succeeded in covalently attaching TPE-ITC to CS and used the resultant TPE-CS for long-term cell tracking [41]. Li and co-authors synthesized TPE-CS NPs by an ionic gelation method using TPE-CS conjugates [44]. By adding tripolyphosphate (TPP) solution dropwise into TPE-CS acetic acid aqueous solution under stirring, TPE-CS NPs can be obtained. As another example, PEG-EP3 polymer synthesized by ring-opening crosslinking PEGylation of an AIE epoxy monomer (EP3) and a 4-arm PEG-amine can also be used for fabricating AIE NPs simply by dispersing the PEG-EP3 polymer into aqueous solution [43].

Alternatively, AIE dyes with proper functional groups can be incorporated as building blocks into the polymer matrix during polymerization. For example, fluorescent silica NPs can be fabricated by surfactant-free sol-gel polymerization reactions of AIE dye-functionalized siloxanes followed by the reactions with tetraethoxysilane [45].

Recently, a strategy combining nanoprecipitation and photo-crosslinking has also been utilized to prepare small sized AIEgen-based NPs, Figure 3H [12]. AIEgens were firstly modified with oxetane groups, and then coprecipitated with polystyrene-oxetane (PS-OXE) into the initial AIE-NPs with around 15 nm in size. Subsequently, upon UV light irradiation, the oxetane groups of AIE-OXE and PS-OXE in the initial NPs could be photo-crosslinked by cationic ring opening polymerization, and the final compact and ultrastable AIEgen-based NPs were obtained. As the newly generated hydroxy groups from the ring opening polymerization, the zeta potentials of the NPs became more negative after photo-crosslinking. A good colloidal stability of the photo-crosslinked AIE NPs against organic solvent (e.g., acetone) was observed [12].

### 2.3. Functionalization of the AIEgen-Based Nanomaterials

**Figure 4:**
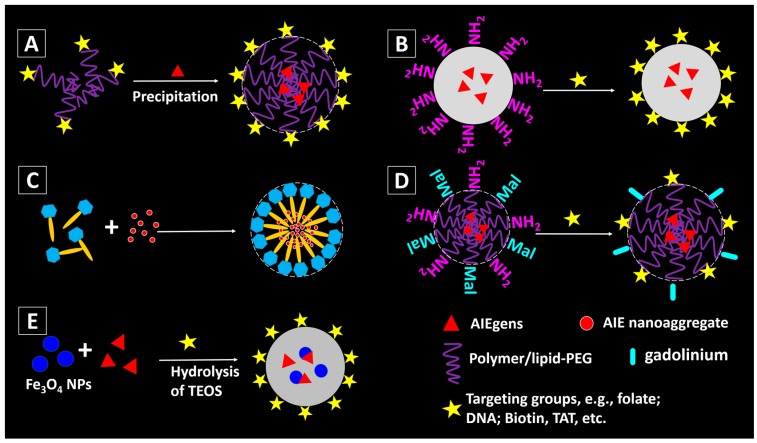
Methods for fabricating functionalized AIEgen-based nanomaterials: (**A**) with pre-modified building matrixes; (**B**) surface modification of the amino groups-decorated AIE/SiO_2_ NPs [36]; (**C**) AIE aggregates can be naturally encapsulated inside the saponin micelles [47]; (**D**) dual functionalization of the AIE NPs by post-modification [48]; (**E**) incorporation of magnetic component (e.g., Fe_3_O_4_) into AIE-SiO_2_ NPs [49].

In order to enhance the targetability, biocompatibility, environmental stability or cell permeability of the nanomaterials, surface modifications/functionalization are usually employed. The targeting efficiency of the nanomaterials can be improved by decorating the nanomaterial surface with bioactive targeting moieties [2,46]. Pre-modification is one way to achieve the nanomaterial functionalization. The targeting molecules can be pre-functionalized to the matrix materials for the encapsulation of the AIEgens. For example, folic acid (folate) can recognize folate receptors which are over-expressed in many cancer cells. Folate modification is therefore a low-cost but effective method for fabricating cancer cells targeting nanomaterials. By precipitating folate modified polymers (e.g., poly([lactide-co-glycolide]-bfolate [ethylene glycol] (PLGA-PEG-folate), lipid-PEG-folate, etc.) with the AIEgens, Figure 4A, fluorescent NPs with AIEgen in the core and folic acid groups on the surface can be obtained [24,32].

Another approach to enable surface decoration is post-modification, Figure 4B,C. For AIE-SiO_2_ NPs, amine groups can be introduced to their surface by reacting with 3-aminopropyltriethoxysilane (APTES), and consequently enabling the NPs to undergo an amidation reaction with the molecules with carboxylic acid groups, Figure 4B. Nucleolin-specific DNA aptamers can be covalently linked on the SiO_2_ NPs with amino groups through two step reactions: first, sulfosuccinimidyl-4-(*N*-maleimidomethyl)-cyclohexane-1-carboxylate (Sulfo-SMCC) was introduced to convert the amines to thiol-reactive maleimides on the NPs; second, thiol-modified DNA aptamers specific to nucleolin were coupled to the NPs via the thiol-maleimide reaction [36]. Likewise, AIEgen-based NPs with amino groups can be covalently linked with a cell-penetrating peptide HIV-1 transactivator of transcription (Tat) protein through carbodiimide-mediated coupling, yielding Tat-AIE dots (Figure 4B).

A method for efficient delivery of the AIE nanoaggregates has been proposed by Tang, et al. recently [47]. As shown in Figure 4C, the AIE NPs are pre-fabricated and then loaded into saponin micelles. They proved that by encapsulating AIE NPs within saponin, a class of naturally occurring bioactive and biocompatible amphiphilic glycosides produced by plants, the obtained AIE NPs can enter the HeLa cells within seconds.

Multi-functionalization can be achieved by introducing two or more valuable components into the NPs (Figure 4D). For example, the AIEgen-based NPs can be modified with both targeting molecules (e.g., Tat) and magnetic gadolinium (e.g., Gd), enabling the high resolution magnetic resonance imaging (MRI) with efficient targeting ability (Figure 4D) [48]. In addition, incorporation of other functional particles into the AIEgen-based NPs can also be used for multi-modality imaging. Core-shell structured AIE-SiO_2_-Fe_3_O_4_ NPs were fabricated by the surfactant-free sol-gel reaction of TEOS and silole-APS catalysed by NH_4_OH in the presence of citrate coated magnetite NPs (Figure 4E) [49]. The formed NPs show both fluorescent and magnetic property, which could enable the fluorescent and MRI imaging [50]. Further modification of the AIE-SiO_2_-Fe_3_O_4_ NPs can be achieved by adding other siloxanes into the reaction mixture, or decorating their surfaces with targeting agents, etc. The details of bio-applications of such multi-functional AIEgen-based NPs will be discussed in the next section.

## 3. Biological Applications

AIEgen-based fluorescent materials demonstrated strong emission, high photostability, excellent biocompatibility, and feasibility for surface functionalization. To date, the AIEgen-based nanomaterials have been extensively exploited for various bio-applications.

### 3.1. Bioimaging—In Vitro and In Vivo

#### 3.1.1. Cell Imaging

**Figure 5:**
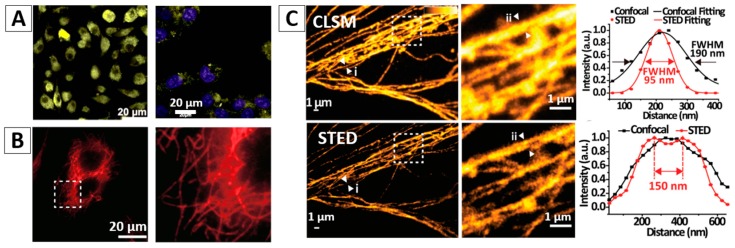

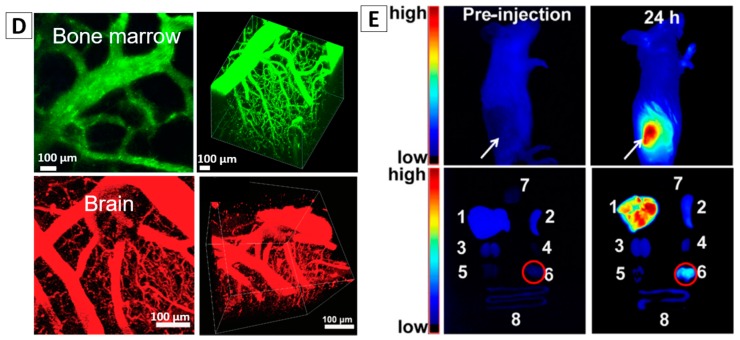
(**A**) Non-targeting cell imaging via organic An18-F127 NPs [52] (left panel) and targeting cancer cell imaging of folate-functionalized AIE-SiO_2_ NPs (right panel) [51]; (**B**) targeted imaging of microtubules in HeLa cells labelled with the Red-AIE-OXE NPs [12]; (**C**) CLSM and STED imaging of the microtubule structures with Red-AIE-OXE NPs and the corresponding line profile of the position indicated by the arrow heads (i) and (ii) [12]; (**D**) In vivo two-photon imaging of bone marrow blood vessels [63] (up) and 3TPFI imaging of brain blood vessels (down) of mice treated with AIE NPs [64]; (**E**) AIEgen-based NPs for non-invasive fluorescence imaging of tumor-bearing mice and the dissected tumors and organs (The white arrows indicate tumor sites and the red circles indicate dissected tumors. 1—Liver, 2—Spleen, 3—Kidney, 4—Heart, 5—Lung, 6—Tumor, 7—Brain, 8—Intestine) [65]. Reprinted with permission from ref. [12,51,52,63,64,65], respectively. Copyright 2017 Wiley-VCH, Copyright 2013 The Royal Society of Chemistry, Copyright 2013 The Royal Society of Chemistry, Copyright 2013 Wiley-VCH, Copyright 2017 The Royal Society of Chemistry and Copyright 2016 Elsevier, respectively.

Due to the low contrast of the cells, contrast agents are usually needed for cell imaging. AIEgen-based nanomaterials have been successfully demonstrated as good contrast agents for cell imaging. For example, fluorogens An18 (derivatized from 9,10-distyrylanthracene with an alkoxyl endgroup) can be encapsulated in the surfactant Pluronic F127, forming An18-F127 nanoaggregates. After incubating the An18-F127 with A549 cells for 3 h, the NPs were internalized into the cells and located at the cell cytoplasm, showing bright yellow fluorescence, Figure 5A (left panel) [51,52]. With proper decoration of targeting molecules, such as biotin, folate, DNA aptamer, antibody, etc., the modified AIEgen-based NPs would selectively target the specific cells as pre-designed [36]. Figure 5A (right panel) shows that the cellular cytoplasm of HeLa cells was easily stained with folate modified AIEgen NPs (9,10-distyrylanthracene entrapped in silica matrixes) [51]. Recently, Fang and the co-authors successfully prepared small and bioconjugated AIE NPs and realized the subcellular imaging using antigen-antibody specific recognition [12]. In their study, three types of AIE-OXE NPs with blue, green, and red emissions decorated with streptavidin were used to stain biotin labelled microtubules in HeLa cells with high selectivity. Microtubules labelled with the Red-AIE-OXE NPs are shown in Figure 5B.

Due to the good photostability, high stimulated emission depletion efficiency and large Stokes shift, AIEgen-based NPs show great potential for stimulated emission depletion (STED) microscopy applications [12,53,54,55,56]. Very recently, people used the above mentioned small sized, ultrastable and highly bright Red-AIE-OXE NPs for microtubule imaging using STED nanoscopy. Compared with confocal laser scanning microscopy (CLSM) imaging, the spatial resolution of STED imaging with Red-AIE-OXE NPs is significantly enhanced to ~95 nm, Figure 5C [12].

#### 3.1.2. Vascular Imaging

Fluorescent imaging of vascular structures is an emerging modality for the diagnosis of many diseases such as cardiovascular diseases and cancers [57]. It shows great potential for monitoring vascular structural change or dysfunction [58,59,60,61]. As mentioned above, AIEgen-based NPs are stable, bright and biocompatible, and thus are ideal for vascular imaging [62].

Liu et al. [63] synthesized 4,7-bis[4-(1,2,2-triphenylvinyl)phenyl]benzo-2,1,3-thiadiazole (BTPTEBT, AIEgen)-loaded lipid-AIE NPs via a modified nanoprecipitation method using as an encapsulation matrix 1,2-distearoyl-sn-glycero3-phosphoethanolamine-*N*-[methoxy(polyethylene glycol)-2000] (DSPE-PEG2000). Two-photon fluorescence imaging (TPFI) results of blood vessels showed that the vessels stained by BTPTEBT-loaded lipid-AIE NPs with green emission were approximately ten times higher than that by Qtrackers 655 and no photo-blinking phenomenon was observed. As shown in Figure 5D, the major blood vessels as well as the small capillaries in the mouse bone marrow could be clearly visualized under the intravital two-photon fluorescence microscope. The 3D reconstruction image (Figure 5D) indicated that AIEgen-based NPs can serve as a safe and effective contrast agent to specially visualize blood vessels using TPFI up to a depth of about 100 mm. B. Liu also demonstrated the feasibility of TPE-FN nanocrystals to visualize blood vessels in mouse ear and skin [29].

Three-photon fluorescence imaging (3TPFI) with NIR excitation is with deep tissue penetration ability, high resolution, and good signal-to-noise ratio (SNR) [64]. A type of mPEG5000-DSPE encapsulated TPEPT NPs (TPE as a donor and [1,2,5]thiadiazolo[3,4-c]pyridine(PT) as an acceptor) with red emission was synthesized and applied for 3TPFI in vivo vascular imaging of a mouse brain model under 1550 nm fs laser excitation. The major blood vessels as well as the small capillaries in the mouse brain can be clearly seen, Figure 5D (the bottom panel) and a fine three-dimensional (3D) reconstruction demonstrated that a penetration depth of 500 mm was achieved [64].

Liao and Liu also demonstrated the use of AIEgen-based NP for the detection of the blood brain barrier (BBB) damage and inflammation which leads to higher permeability of blood vessels [66]. AIEgen-based NPs with different sizes (TTF coprecipitated with DSPE-PEG) were used to evaluate BBB integrity and map vascular leakage in a rat model, for which photothrombotic ischemia (PTI) was selected to induce the BBB damage. The experiments demonstrated that the TTF based NPs with a size of 30 nm could provide clear evaluation of vascular leakage at the exact location of brain stroke without nonspecific leakage at other intact regions, achieving higher sensitivity and specificity for the detection of BBB integrity as compared to Evans Blue (EB, the EB-based method is a common technique for the evaluation of BBB damage).

#### 3.1.3. Tumor Imaging

Cancer is one of the most common causes of death worldwide, accounting for a few million deaths per year [67]. Imaging, including fluorescent imaging, is able to provide morphological, structural, metabolic and functional information of tumor, and forms an important part of cancer clinical protocols [68]. AIEgen-based nanomaterials are with excellent optical properties and good biocompatibility which are ideal for in vivo imaging. Their application in tumor imaging has been intensely studied [20,65,69,70].

The fast growing agiogenic blood vessel in tumor tissue is leaky that NPs with a proper size will penetrate and accumulate. The tumor targeting ability of these AIEgen-based NPs mainly relies on passive enrichment of NPs by this enhanced permeation and retention (EPR) effect [65,71]. Tang et al. used BSA-encapsulated TPE-TPA-DCM AIEgen-based NPs for tumor imaging [20]. The NPs are around 148 nm in size, and emit at 668 nm with a quantum yield of 12% at a 3 wt % fluorogen loading. They also possess great photostability, showing 90% of their original fluorescence intensity even after expose to continuous laser scanning for 10 min. Their potential for tumor imaging was examined using a tumor-bearing mouse. Results showed that after intravenous injection of the NPs into the tumor-bearing mouse, intense fluorescence on the tumor site, which was in sharp contrast to other parts of the mouse body, can be observed. Similarly, Zhang and the co-authors fabricated DSPE-PEG2000 micelles encapsulated bis(4-(*N*-(2-naphthyl) phenylamino) phenyl)-fumaronitrile (NPAPF) with an average size of 65 nm for tumor imaging [65].

#### 3.1.4. Dual-Modality Imaging

It is well accepted that single imaging techniques are sometime insufficient for accurate diagnosis [72]. Multi-modality imaging techniques could combine the advantages of more than one imaging modality, and thereby have attracted increased interests in recent years [73,74,75]. For example, fluorescent imaging presents advantages in the visualization of tumors and related biomolecules in vivo with high sensitivity, but it also suffers from limited penetration depth even at Red-NIR wavelengths [65]. In contrast, MRI and CT techniques are superior with excellent spatial resolution, while the low sensitivity is their main limitation [74,76]. Fluorescence/MRI or fluorescence/CT dual imaging overcomes the limits of the other and provides complementary information for accurate tumor diagnosis in vivo, showing great potential in biological application [74,77].

**Figure 6:**
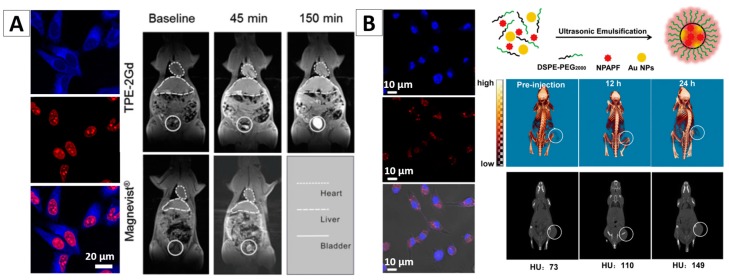
(**A**) Cell imaging and in vivo MIR imaging with TPE-2Gd NPs [78]; (**B**) Molecular structures of co-encapsulation of NPAPF and Au by DSPE-PEG2000 and their application in fluorescent cell imaging and in vivo CT imaging [65]. Reprinted with permission from [65,78], respectively. Copyright 2016 Elsevier and Copyright 2014 American Chemical Society, respectively.

AIEgen-based nanomaterials incorporated with another type of imaging agents would enable dual-modality imaging. Tang and co-workers fabricated a fluorescence/MIR dual-modality imaging NPs (TPE-2Gd), which were developed by the covalent conjugation of one TPE unit with two Gd diethylenetriaminepentaacetic acid units [78]. The TPE-2Gd is an amphiphilic AIE molecules, so it can easily form micelles with a diameter of ~165 nm when its concentration reaches a certain level in an aqueous medium. MTT assays proved that the TPE-2Gd possesses good biocompatibility. It can enter cancer cells and stain their cytoplasmic regions with blue fluorescence, Figure 6A. For in vivo experiments, TPE-2Gd or the commercial contrast agent Magnevist with concentration of 0.1 mmol/kg Gd^3+^ was intravenously injected into mice. After injection, a strong signal in the heart was observed. The signal of Magnevist in the heart returned to the baseline within the first 60 min. TPE-2Gd showed a long intravascular half-life time (up to 1 h), which was five times longer than that of Magnevist. This could be attributed to a low filtration rate of the TPE-2Gd nanoaggregates. Long intravascular half-life time can provide more time for diagnosis and treatment. Figure 6A indicated that the TPE-2Gd NPs significantly enhanced the contrast of the liver and are promising for the discrimination of the lesion and normal tissues. The signal in the bladder at 150 min after injection revealed that the NPs can be excreted through the kidney [78].

In another study, Zhang et al. developed one type of fluorescent NPs that used DSPE-PEG2000 to encapsulate both AIEgen (NPAPF) and gold NPs, Figure 6B [65]. NPAPF is an AIEgen with far-red/near-infrared (FR/NIR) emission, and the gold NPs is a typical contrast agent for CT imaging. Hence this kind of combination is expected to enable fluorescence/CT dual modality imaging. The formed fluorescent NPs with NPAPF/gold (i.e., NPAPF/Au-AIE NPs) were with an emission peak at ~640 nm, a Stokes shift of ~120 nm, and a quantum yield of ~8%. As gold NPs have significant fluorescence quenching effects for fluorescent agents, it is hard to maintain the fluorescence property under the existence of a high concentration of gold NPs. However, attributing to the AIE nature, NPAPF can significantly counteract this quenching effect and thereby ensure the high quantum yield of the hybrid NPs. Experiments demonstrated that the hybrid NPs are with a low cytotoxicity to both tumor cells and normal cells even at relatively high concentration. Figure 6B shows their potential for both fluorescent and CT imaging. These fluorescence/CT dual-modal images could provide both high sensitivity and 3D information with anatomical resolution, which may greatly aid precise cancer diagnosis [65].

Photoacoustic (PA) imaging is an emerging imaging modality based on acoustic detection of optical absorption [74,79,80,81]. Recently, various AIEgens with broad NIR light absorption have been developed, which could serve as both efficient fluorescence and PA imaging agents [76,82]. Liu and co-workers explored the possibility for AIEgens to be used in PA imaging [76]. They found that the PA signal of conventional fluorophores such as 2,3-bis[4-(diphenylamino)-phenyl]fumaronitrile (TPAFN) could be significantly enhanced through conjugation with TPE. Integration of TPE (T), triphenylamine (T), and fumaronitrile (F) unites yielded adduct TTF. TTF showed 170% higher PA signal as compared to that of TPAFN. But there was a significant reduce in PA signal for TTF in NPs due to the inhibition of intramolecular motions [76]. More recently, in order to achieve promising PA signal of solid NPs, the method of enabling active intramolecular motions in solid state by introducing molecule with propeller structure was proposed by Liu, et al. [83]. Two TPE units (propeller structure, electron donor) were introduced to 4,9-di-(5-bromothiophen-2-yl)thiadiazoloquinoxaline (TTQ, electron acceptor), to form a D-A-D configuration (BTPETTD). The BTPETTD NPs were formed by encapsulating them into DSPE-PEG2000 matrix, showing obvious fluorescence upon excitation at 700 nm. Attributed to the free rotation of their propeller structure, such NPs showed excellent PA signal output which was higher compared to the conventional gold nanorods (50% increase) and the NPs with TTQ cores without TPE modification (15% increase). For example, with a concentration at 100 µg/mL, BTPETTD NPs displayed ~820 a.u. of PA signal, while the gold NPs only showed ~400 a.u. of PA signal. In vivo experiment demonstrated their potential for high-resolution PA imaging of sentinel lymph node of rats.

### 3.2. Cell Tracing

Long-term cell tracing could help to elucidate cell behavior during cancer metastasis or tissue regeneration [1,84,85]. Compared with cell imaging, cell tracing, especially long-term cell tracing, has a higher requirement for the labeling reagents. The ideal labeling reagents for this purpose should efficiently label the cells of interest, well retain inside the cells for a relatively long period of time, provide a bright and stable signal under low/moderate light irradiation, and be with excellent biocompatibility. Most of the present fluorescent probes proposed for long-term cell tracing have inherent drawbacks, such as the heavy metal element induced high cytotoxicity from semiconducting QDs [86], aggregation-caused quenching for carbon dots [3], tedious transfection process of the fluorescent protein [87], etc. Generally speaking, AIEgen-based nanomaterials are with the high emission efficiency, low toxicity, good photostability and large Stokes shift, and therefore are ideal candidates for non-invasive long-term in vitro and in vivo cell tracing.

**Figure 7:**
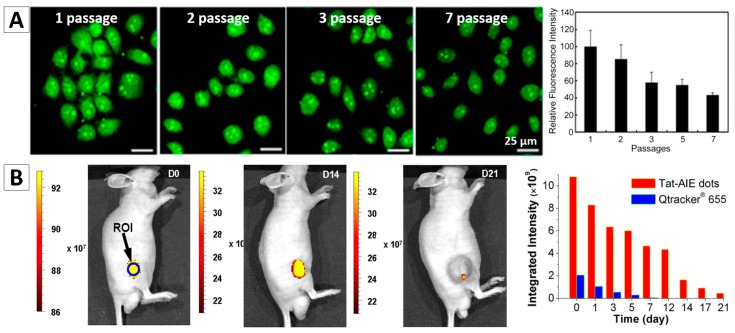

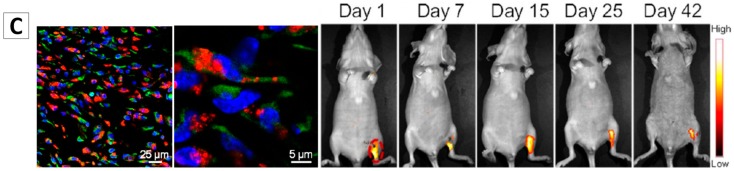
(**A**) HeLa cells traced by TPE-NSCS NPs at different passages and the fluorescence intensity of TPE in HeLa cells stained with TPE-PNIPAM at different passages [88]; (**B**) Long-term in vivo tracing for 21 days via labelling cells with AIE NPs [30]; (**C**) Tracking of ADSCs in vivo at single-cell resolution and representative time-dependent in vivo fluorescence images of the ischemic hind limb-bearing mouse that was intramuscularly injected with AIE dot-labeled ADSC-containing Matrigel [89]. Reprinted with permission from ref. [30,88,89], respectively. Copyright 2013 Nature, Copyright 2015 American Chemical Society and Copyright 2014 American Chemical Society, respectively.

It was reported that the temperature sensitive organic NPs with AIE feature, tetraphenylethene-based poly(*N*-isopropylacrylamide) (TPE-PNIPAM) NPs, can be used as a probe to trace HeLa cells for a long term, Figure 7A [88]. Because the PNIPAM is a temperature sensitive polymer, which has a lower critical solution temperature at 32 °C (LCST) in aqueous media. When the temperature reaches to the LCST, PNIPAM undergoes a transition from hydrated coil to dehydrated granule. Hence, the size and fluorescence intensity of the NPs synthesized from TPE-PNIPAM can be tuned by varying the environmental temperature. For example, the fluorescent intensity decreased if increasing temperature, as it can quicken the molecular motions and intramolecular rotations. For long-term tracing study, the HeLa cells were stained with TPE-PNIPAM for 24 h, and then were repeatedly passaged every 2 days and measured by confocal microscope and flow cytometry. The stained cells can retain the strong fluorescence intensities up to 15 days, and over 40% of fluorescence remained for as long as seven passages (Figure 7A), which revealed that the TPE-PNIPAM NP is a good candidate for long-term cell tracing.

In vivo long-term cell tracing using AIEgen-based NPs has been intensely investigated in recent years. Liu and Tang et al. encapsulated two AIE dyes into lipid PEG, and then synthesized cell penetrating peptides (Tat) modified PEG-AIE NPs, named Tat-PEG-TTF NPs [30]. The in vivo study indicated that Tat-PEG-TTF NPs are better than the commercial QDs Qtrackers 655 in terms of the cell tracking performance. The C6 glioma cells labelled with the Tat-PEG-TTF NPs can be tracked for 21 days while the cells stained by the commercial Qtrackers 655 can only be traced for no more than 7 days (Figure 7B). Liu, Tang and Kong, et al. developed one type of AIE NPs for long-term tracking of adipose-derived stem cells (ADSCs) and their regenerative capacity in living mice, Figure 7C [89]. AIEgen NPs-labelled stem cells were applied to wounds on mice. The bright fluorescence from the labelled cells allowed us to monitor the cells for over a period of 42 days, enabling the study of stem cells migration and distribution during cell therapy.

### 3.3. Theranosis

#### 3.3.1. Photodynamic Therapy (PDT)

PDT is a non-invasive method for controlled removal of the tumor cells [2]. The mechanism of PDT is that the photosensitizers (PSs) are capable of undergoing photophysical and photochemical processes upon light irradiation to generate toxic reactive oxygen species (ROS) in situ [90,91]. It is believed that the intersystem crossing occurs from the singlet excited state to the triplet state upon excitation of the PSs, followed by reaction with molecular oxygen to yield ROS, including singlet oxygen (^1^O_2_) [92]. The resultant toxic ROS will further induce cell death. Some AIEgens have recently been demonstrated as superior PSs. Generally, the well-designed AIEgen-based NPs can serve both as a targeted fluorescence imaging contrast agent and a photosensitizer used as PDT agent [92,93].

**Figure 8:**
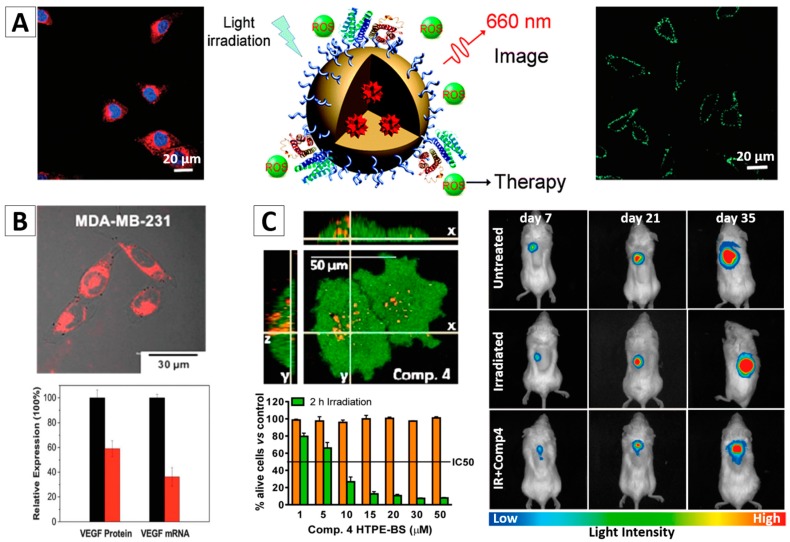
AIE NPs with cRGD (**A**) and siRNA (**B**) functionalized surface for targeted imaging and image-guided photodynamic therapy (PDT) [92,94]; (**C**) CLSM images of PC3 cell after an overnight incubation with TPE-NPs of Comp 4 (TPE-benzothiazole conjugates hexyl); Concentration dependent cytotoxicity of Comp 4 in PC3 cells in dark (orange bars) and light (green bars) conditions; and representative non-invasive bioluminescence images of PLuc-expressing PC-3 tumors [93]. Reprinted with permission from [92,93,94]. Copyright 2014 The Royal Society Chemistry, Copyright 2016 American Chemical Society and Copyright 2016 The Royal Society Chemistry, respectively.

In one study, a tumor-targeting bioactive tripeptide, cyclic Arg-Gly-Asp(cRGD) peptide, was conjugated with TPE-EPA-DCM-lipid-PEG AIE NPs (cRGD TPE-EPA-DCM AIE NPs). The formed NPs were highly selective to the α_v_β_3_ integrin-positive cells (e.g., MDA-MB-231). Figure 8A (left panel) shows strong red fluorescence signal of the MDA-MB-231 cells stained by the cRGD TPE-EPA-DCM AIE NPs [92]. When irradiated with light (0.25 W/cm^2^) for 1–2 min, ROS was efficiently generated by the fluorescent NPs in MDA-MB-231 cells as indicated by the bright green emission from a ROS sensor. (Figure 8A, right panel). The cRGD TPE-EPA-DCM AIE NPs were non-toxic even to integrin-positive MDA-MB-231 cells in the dark environment. After light illumination, a significant decrease of cell viability to less than 15% in 24 h was observed. Fluorescein isothiocyanate (FITC)-tagged Annexin V staining indicated that the exposure of light irradiation to the MDA-MB-231 cells containing cRGD TPE-EPA-DCM AIE NPs led to cell death through an apoptotic pathway (Figure 8A, right panel). The control experiment showed that the targeting effect of TPE-EPA-DCM AIE NPs and the PDT effect was less significant to the integrin negative cells (MCF-7 cancer cells and NIH 3T3 normal cells with low α_v_β_3_ integrin expression) [92].

More recently, based on the above discussed study, Liu and Li developed an AIEgen-based multifunctional organic NPs with siRNA decoration, which can be used for targeted PDT and RNA interference therapy, Figure 8B [94]. The same AIEgen, TPE-EPA-DCM, was used in this system. It was proved that the cancer cells stained by such NPs showed bright fluorescence, and ROS could be effectively generated upon light irradiation. The VEGF siRNA on the surface of NPs can be successfully transfected to the cancer cells and thus to downregulate VEGF mRNA and protein expressions. Cell viability studies demonstrated that, due to the synergistic effects between PDT and RNA interference, the siVEGF-TPE-EPA-DCM NPs could selectively and efficiently kill the α_v_β_3_ integrin overexpressed cancer cells [94].

Investigations on the in vivo PDT treatment of tumors have also been reported [6,74]. AIE NPs based on TPE with a size of ~10 nm were synthesized by Ramaiah and co-workers, and were applied for PDT study, Figure 8C [93]. These NPs exhibited a rapid cellular uptake, negligible dark toxicity, significant ROS generation efficiency, and high in vitro photocytotoxicity as well as in vivo photodynamic activity. This study demonstrated that such AIEgen-based NPs have great potential for advanced PDT in vivo for cancer treatment.

#### 3.3.2. Drug Delivery

In recent years, researchers have put great efforts on studying the distribution of the drug carriers and the drug release process. With the assistance of fluorescent agents, drug carriers and/or the drugs can be labelled and tracked. Various studies reporting successful examples of employing the AIEgen-based nanomaterials as drug carriers for in vitro and in vivo studies are available to refer to [95,96,97,98].

**Figure 9:**
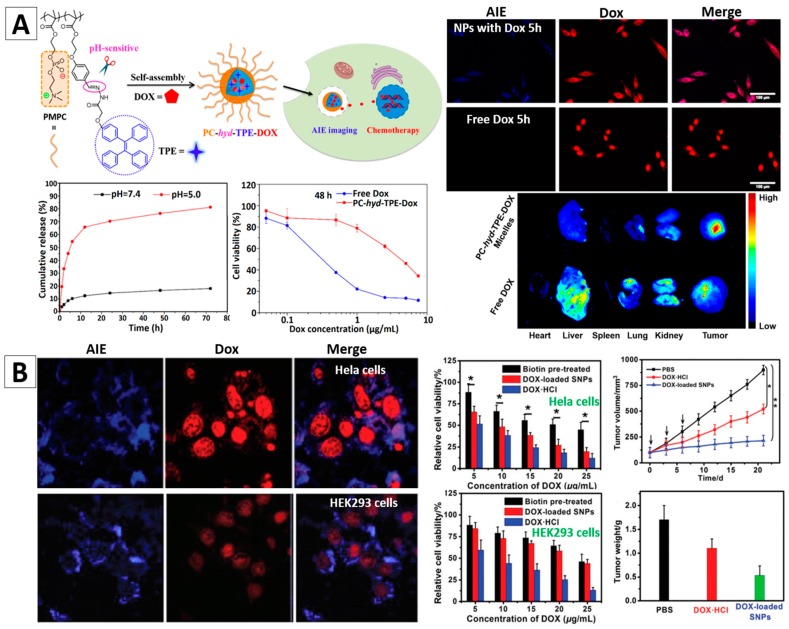
(**A**) PC-hyd-TPE micelles for combined AIE active imaging and pH-responsive drug delivery and release (in vitro and in vivo). Fluorescence images of HepG2 cells co-cultured with the DOX loaded micelles or free DOX for 5 h (top-right) and fluorescence images of excised tissues at 6 h post-injection of the DOX loaded micelles and free DOX (bottom-right) [95]; (**B**) HeLa and HEK293 cells incubated with DOX-loaded AIE-SNPs for 4 h; Cytotoxicity of HeLa and HEK293 cells with different treatments; Tumor growth inhibition curves on the HeLa tumor model after various formulations (* *p* < 0.05) and the average weight of the tumors of mice bearing HeLa tumors after different treatments [99]. Reprinted with permission from [95,99], respectively. Copyright 2016 American Chemical Society and Copyright 2016 The Royal Society Chemistry, respectively.

Jin and co-workers reported one type of pH-responsive micelles with AIE features for doxorubicin (DOX) delivery, Figure 9A [95]. They used a novel zwitterionic copolymer poly(2-methacryloyloxyethylphosphorylcholine-co-2-(4-formylphenoxy)ethyl methacrylate) (poly(MPC-co-FPEMA)) and TPE to form PMPC-hyd-TPE copolymers via acid-cleavable hydrazone bonds. Spherical PC-hyd-TPE micelles can be fabricated by these copolymers. DOX can be easily encapsulated in the micelles, and be freed via tuning pH, as illustrated in the release curves in Figure 9A. The in vitro experiments revealed that the HepG2 cells stained by PC-hyd-TPE micelles showed high-quality fluorescence feature. The PC-hyd-TPE-DOX micelles could be efficiently internalized by HepG2 cells, and showed a remarkable cytotoxicity to the cells with IC_50_ of 4.37 and 0.96 μg/mL after 48 and 72 h, respectively (Figure 9A). Ex Vivo fluorescence imaging results revealed an enhanced accumulation and DOX delivery of PC-hyd-TPE-DOX micelles in tumor tissue [95].

Huang et al. reported AIEgen (TPE)-based supramolecular NPs (SNPs) synthesized from AIE-active amphiphilic supramolecular brush copolymers (SBPs), which were built based on a novel pillar [5] arene-based host-guest molecular recognition and synthesized by using the PTPE (polystyrene with 4,4′-bipyridinium derivative and TPE groups) and P5-PEG-biotin as building blocks [99]. The core-shell SNPs (P5-PEG-biotin-PTPE) can be utilized as a nanocarrier to entrap DOX. The biotin on the surface could make them prefer to target to cancer cells. As illustrated in Figure 9B, blue fluorescence arising from PTPE and red fluorescence from DOX were observed in HeLa and HEK293 cells after incubation with the P5-PEG-Biotin-PTPE SNPs for 4 h (Figure 9B). Cancerous HeLa cells with higher expression of biotin receptor showed increased uptake of AIE-SNPs compared with HEK293 cells. Therefore, the cancer cells were better stained and the AIE-SNPs had a selective cytotoxicity towards cancer cells over normal cells. Their cancer therapy effects were further confirmed by the in vivo experiments using HeLa tumor bearing mice, Figure 9B. The above exemplified studies demonstrated that the AIEgen-based nanomaterials could serve as effective theranostic nanoplatforms with the combined merits of high-contrast cancer diagnosis and drug delivery features.

## 4. Conclusions and Perspectives

Compared with conventional organic fluorophores, which show reduced emission at a high concentration, the AIEgens display intense emission in condensed phases. AIEgens can be incorporated into nanomaterials either by non-covalent binding or by covalent binding strategies. The surfaces of the formed AIEgen-based nanomaterials can be facilely modified with functional molecules such as peptides, proteins, etc. to enhance their targetability and/or cell permeability. Thanks to their high brightness, excellent photostability and nice biocompatibility, the AIEgen-based nanomaterials are advantageous for biological applications, especially for bioimaging, long-term cell tracing, and cancer diagnostics and therapy.

Even though tremendous efforts have been directed to the area of AIEgen-based nanomaterials, it is still a quite young research field with challenges as well as opportunities [100]. Here we list a few research directions that may attract more attention and interest in the future: (1) ultra-bright and stable AIEgen-based NPs with far-red/near-infrared emission for bioimaging applications. To enable bioimaging with a deeper penetration depth, bright AIEgen-based NPs with longer absorption and emission wavelength are highly desired; (2) ultrafine AIE nanocrystals with controllable size and morphology. AIEgen-based nanocrystals show great optical properties. Fluorophores with crystal induced emission properties or room temperature phosphorescence properties also have great potential for bio-applications if we manage to fabricate them into nano-sized crystals. Previous reports reveal that AIE crystals with small size are difficult to obtain. New methods and techniques for well control the nanocrystal size and morphology are greatly needed; (3) AIEgen-based nanomaterials as sensors. AIE-active molecules for sensing purposes have been widely investigated, however, there are few reports about sensors employing the AIEgen-based NPs. We believe that more AIEgen-based responsive nanomaterials will be designed and developed as nano-biosensors, especially for clinical diagnostics. In addition, to promote the successful implementation of AIEgen-based nanomaterials for practical biological application, more studies on understanding the mechanism of the uptake, distribution and clearance of the AIEgen-based NPs at cellular and organism level are necessary.

## Figures and Tables

**Table 1:** Optical properties of typical AIEgens.

AIEgens	λ*_Ex_* (nm)	λ*_Em_* (nm)	Quantum Yield
TPE [26]	299	462 (solid aggregate)	49% in film state
TPEPy [21]	350	468 (film)	100% in film state
BTPETD [27]	350	539 (film)	89% in film state
HPS [22]	363	497 (film)	78% in film state
TPE-OXE [12]	365	620 (solid powder)	57% in solid state
TPE-EPA-DCM [20]	480	633 (THF/water mixture)	12% in BSA matrix with a loading ratio of 3.07% TPE-EPA-DCM
TTF [28]	500	660 (THF/water mixture)	52.5% in solid state
